# Leave or stay? A narrative inquiry of tensions in novice English teachers’ professional identity construction at China’s private universities

**DOI:** 10.3389/fpsyg.2025.1485747

**Published:** 2025-02-03

**Authors:** Xiangchen Zhang

**Affiliations:** 1 School of Humanities and International Studies, Zhejiang University of Water Resources and Electric Power, Hangzhou, China; 2 The School of Curriculum and Pedagogy, The Faculty of Arts and Education, The University of Auckland, Auckland, New Zealand

**Keywords:** narrative inquiry, novice English teacher, professional identity construction, tensions, private university

## Abstract

Novice English teachers at China’s private universities are easily confronted with tensions when they constructed the professional identity in the early career stage. Drawing on a theoretical framework of social identity theory, self-discrepancy theory and situated learning theory, this narrative inquiry study explored the causes, manifestations, and solutions to teachers’ professional identity tensions through in-depth interviews. Data were interpreted through metaphorical biographies and thematic analysis. Results show that tensions were caused by discrepancies among three self-concepts and conflicts between the English teacher identity and the private university teacher identity. In tensions, many aspects of participants’ professional identities were impaired and they unfulfilled the organizational identification. Participants reconciled three selves and adopted two approaches (assimilation and dissimilation) to navigate PI tensions. Policy makers are suggested to offer a well-established identity policy and sufficient research funding to facilitate the academic development of both private universities and teachers there. Private universities are suggested to provide diverse supports to teachers for their career development. Novice teachers could take the initiative of adapting to the culture and regulations of private universities and combine their research interest with the working context.

## 1 Introduction

Teachers’ professional identity (PI) construction is a socialization process of their personal cognition (Le Maistre and Paré, 2010) influenced by contextual and sociocultural factors (Gibbons, 2020). For novice English teachers, the beginning 3 years of the academic career (Baldwin and Blackburn, 1981), especially the first year (Zhukova, 2018), is critical in their professional learning and PI construction. In this period, they might suffer from PI tension or crisis (Vanegas et al., 2021). It marks a “transitional and bifurcation point” (Sadovnikova et al., 2016, p. 6899) and a major challenge in their professional identification triggered by the conflict between teachers’ expectations of professional images and institutional requirements for them (Beijaard et al., 2004). Whether they could solve the conflict or not determines their retention or resignation (Fenwick, 2011; Lee et al., 2013). So, the navigation of tensions is pivotal for teachers’ PI construction (Pillen et al., 2013).

This study aims at exploring tensions in novice English teachers’ PI construction at China’s private universities because it was noteworthy and complicated but rarely analyzed (Zhang et al., 2021; Zhou et al., 2018). The complexity of their PI construction can be attributed to two contextual features inherent in their PIs.

First, private university teachers felt that their professional competence was underestimated by the public due to the marginalized status and the poor reputation of private universities among Chinese tertiary educational institutions (Zhou et al., 2018). The negative reputation was derived from a substandard education quality and a low level of administrative efficiency (Ozturgut, 2011) that led to a problematic working context where teachers’ survival and development were restricted (Wang et al., 2020).

Second, new professional demands on English teachers emerged with reforms of English language teaching (ELT) and the English major. This study is contextualized in China’s tertiary education field, which is influenced by international trends of ELT. Globally, ELT has gone through remarkable changes over the past 20 years marked by two prominent trends since the 1980s: neoliberalism and neo-managerialism (Barkhuizen, 2009; Jin and Cortazzi, 2006). They caused an enormous reform of China’s ELT policies and a drastic transformation of English as a discipline (Chen et al., 2020; Jenkins, 2019). In universities, teachers’ self-supervision was emphasized, and their salaries were directly related to performance reviews (Huang and Peng, 2015). Researchers were transforming from traditional scholars to “deeply socialized professionals” (Huang and Peng, 2015, p. 47) who were required to produce academic research to acquire economic benefits for universities and make universities more competitive in academia worldwide. This commercialisation of academic achievements brought pressure to teachers’ research abilities and further led to their PI tensions (Shumar, 2013).

Based on the research aim and significance, three research questions were proposed:

1. What are causes of tensions in the PI construction among novice English teachers in China’s private universities?
2. What are manifestations of those tensions?
3. What are solutions to those tensions?

The three research questions covering three topics (causes, manifestations, and solutions) are sorted in accordance with the chronological order of the occurrence of participants’ critical incidents (Megawati et al., 2020). This study attends to three sections of teachers’ identity formation processes: antecedent, incident and outcome (Chell, 2004; Holloway and Schwartz, 2014) and aims to explore the dynamic, complex, and variable process of identity reconstruction from sociocultural and psychological perspectives.

## 2 Literature review

Teacher PI development is a pivotal dimension of the teaching profession. Teacher PI development is influenced by the social, emotional issues (Arroyo, 2021), psychological, academic environmental factors (Kovalčikienė and Bukšnytė-Marmienė, 2021), pedagogical beliefs (Cheng, 2021; Sang, 2023), and obstructions in normative contexts (Tokoz Goktepe and Kunt, 2021). Chen et al. (2022) highlighted the reciprocal interaction between emotions and professional identities of student teachers. Teachers could develop and enhance their PIs through co-teaching experiences (Valdés-Sánchez and Espinet, 2020), digital storytelling (Kim et al., 2021), electronic collaborative discussion forums (Ghamoushi and Mohamadi Zenouzagh, 2020), effective supervision (Chaula, 2024), improved teacher education curricula (Al Juhaish et al., 2020a), reflective practices (Liu, 2024).

The PI development of EFL teachers is crucial for their career growth and success. Various studies have explored both internal and external factors that contribute to the formation and evolution of EFL teachers’ professional identities.

Some studies focus on internal factors. EFL teacher PI development has the dynamic nature. Individual professional agency is essential in expressing PI, developing professional relationships (Green and Pappa, 2021), ensuring the sustainability of professional development, and avoiding teacher attrition (Huang, 2021). Ulla (2024) investigated early-career EFL teachers’ engagement in professional learning, focusing on teaching beliefs and professional values as key factors in professional development. Through the lens of the possible selves theory, Cosgun and Savaş (2023) found that different teacher groups had similar perceptions of ideals and fears, and emphasized factors like professional development and language proficiency. A data-driven model for EFL teacher PI development was proposed.

Other studies highlight external factors such as the teaching environment, professional settings and evaluation systems. Al Juhaish et al. (2020b) traced three Saudi EFL teachers and generated four themes through classroom observations and in-depth interviews: language-related identity, context-related identity, teaching skills, and community of practice membership. Tavakoli (2021) emphasizing the pivotal role of textbooks in shaping teachers’ content knowledge. Mehdizadeh et al. (2023) found that EFL teachers developed PIs by realigning possible selves in evolved community of practice (CoP). Ardi et al. (2023) explored how appreciative collaborative reflection catalyzed the configuration of Indonesian EFL teachers’ professional identities during a teacher professional education program. The qualitative study of Shafiee Rad (2023) showed that a accountability-focused evaluation system can be harmful to teachers’ PIs, and there is a significant relationship between teachers’ identity and their well-being, effectiveness, commitment, and sense of agency.

There are no major contradictions in the existing literature about EFL teacher PI development. Although there are substantial studies exploring different aspects and influencing factors of teacher’s PIs in various contexts via different research methods, further research is still needed to explore the impact of different educational policies and cultural backgrounds in various regions on EFL teacher PI development.

PI tension or crisis is a common phenomenon among highly accomplished academics such as university teachers who feel a lack of social recognition and belongingness, a sense of incompetence (Vaughn et al., 2020), and doubt many components of their PIs (self-image, teaching competence, attitude, teaching beliefs and values, future expectations of career) (Bolívar et al., 2014). Multiple self-concepts are incompatible within their PIs and they have difficulty reconciliating diverse demands, values, and goals embedded in the commitment to those concepts (Baumeister et al., 1985). Afterward, they negotiate and reconcile their multiple roles by varied forms of participation and sense making of the membership of different professional communities (Hodgen and Askew, 2007). Ghiasvand et al. (2023) mapped novice and experienced Iranian EFL teachers’ PI conflicts and confrontation strategies. Through semi-structured interviews and narrative frames with 30 EFL teachers, they found that both novice and experienced teachers faced identity conflicts due to factors like teaching philosophy mismatch, with different sources of conflict for each group, and suggested various confrontation strategies.

Although PI tension is a popular subject in the field of teacher PI (Vanegas et al., 2021), the research of its intricate nature among novice teachers at the tertiary education level is insufficient, especially in the context of private universities in China. To address this gap, this study explores the experiences of tensions in PI construction of novice English teachers at China’s private universities and coping methods of those tensions to bolster their professional development.

In the field of teacher PI, metaphor serves as a powerful conceptual tool. It is both a figure of speech and a cognitive mechanism. Teachers use metaphors to describe and understand their functions, roles, experiences, and identities. In this study, Metaphor is a critical rhetoric device in writing biographies. The purpose of applying metaphors to each biography was to re-describe their teaching lives in a vivid, authentic way that intensifies the presentation of findings (Lakoff and Johnson, 2003; Richardson, 2005). It also aims to resonate with readers who have similar experiences as novice teachers. Through engaging with metaphors, participants’ interpretations of their PI reconstruction, the complex and diverse nature of teacher PI, implicit beliefs and values within the teaching profession were explored.

Many studies interpret teachers’ PIs through their self-created metaphors which are explicit carriers of teachers’ teaching beliefs (Farrell, 2006). Writing metaphors is proved to be an effective method for pre-service teachers to understand their PIs deeply in teacher education programmes (Buchanan, 2015) and develop their views of teaching reality (Lin et al., 2012; Farquhar and Fitzsimons, 2013) and curriculum (Akinoglu, 2017). The interpretation of metaphors is inseparable from contexts (Redden, 2017). It is through metaphorical discourse (Alsup, 2006) that novice teachers communicate their ideas about teaching and structure and intensify their perceptions of PI (Lakoff and Johnson, 2003). Koro-Ljungberg (2001) expanded the knowledge of how Finnish science professors understood creativity by analyzing metaphors presenting in multilayered texts: academic story, personal story, and the meta-story. Despite extensive studies analyzing teachers’ PIs through metaphors created by them, there is a notable absence of studies reinterpreting teachers’ experiences of PI tensions through metaphors written by the researcher. The author retold stories of participants and design generative metaphors (Schön, 1993) to elucidate teachers’ PIs from new standpoints (Cross, 2001).

## 3 Theoretical framework

The theoretical framework comprises three theories at the macro, meso and micro levels. The first theory is social identity theory (Tajfel and Turner, 1986, 2004) from the macro perspective of social structuralism. The terms of social categorization, social identification, and social comparison between in-group and out-group were applied to analyze the causes and manifestations of participants’ PI tensions. The concepts of social change and social mobility were used to explain the solutions to their PI tensions. The second theory is situated learning theory (Lave and Wenger, 1991) from the meso, contextual perspective. It is applied to explain how teachers’ participation in the community of practice (CoP) influenced their PI development. In this study, the CoP refers to a group of EFL teachers at private universities who share a common concern, a set of problems, or a passion about English language teaching. They deepen their knowledge and expertise in this area by interacting on an ongoing basis. CoP is also a place where novice EFL teachers learn to teach, develop a sense of PI, and become competent teachers (Wenger, 1999). The concepts of peripherality, inbound trajectory, peripheral trajectory, and outbound trajectory were used to explain the cause (peripherality) and solution (three trajectories) to teachers’ PI tensions. Third, from the micro, psychological perspective, self-discrepancy theory (Higgins, 1987) is deployed to interpret the cause and manifestation (discrepancies between three conflicting self-concepts) and the solution (reconciliation of three selves) to teachers’ PI tensions.

The three theories are interrelated, complementary to each other and jointly act on data analysis. Social identity theory was combined with situated learning theory because novice EFL teachers belonged to several social or professional groups. Their PIs are also social identities and group identities. They constructed their PIs and encountered the PI tensions in legitimate peripheral participation within their groups (Wenger, 1999) and in social comparison between the in-group and the out-group (Turner, 1975). Psychological theories are additions to social-cultural theories that makes the interpretation of findings integrated because teacher’s PI is a subjective construction of self-concepts in the workplace (Vanegas et al., 2021).

### 3.1 Social identity theory

Social identity theory explains teachers’ identifications within different groups and their perceptions of belonging to various social categories (Robinson and Tajfel, 1996; Tajfel and Turner, 1986, 2004; Turner et al., 2010). When teachers belong to a social group, their self-images become part of the representative images of group members (Falk and Miller, 1998). Those two parts exist in teacher’s beliefs about teaching and change when teachers negotiate with multiple social and cultural contexts and self-evaluation (Hamachek, 1999).

There are three sequential processes of social identity construction: social categorization, social identification, and social comparison. In the first step social categorization (Hogg, 2021; Turner et al., 1987), individuals classify themselves into certain social groups based on distinct characteristics (Bourdieu, 1987). The second step is social identification. Individuals adopt the sense of belonging, self-esteem (Lee and Robbins, 1998), membership, values, and culture within their in-groups (Turner, 1984). In the third step social comparison, individuals compare different traits and qualities of the in-group and out-group (Tajfel, 1981). Those traits include teachers’ competence, professional achievements, the social status and prestige of the universities they work at (Gilbert et al., 1995).

Individuals adopt two different mindsets when they perceive their current social identities as less satisfactory. The selection of mindset is dependent on the social statuses of individuals’ groups and the probability of leaving those groups (Boyer and Lecheler, 2023). If individuals choose the social mobility mindset, they leave their current groups and move to other groups with higher social status; if individuals choose the social change mindset, they strive to improve their current groups’ social statuses to make them more positively distinct from other groups (Tajfel and Turner, 1986).

### 3.2 Situated learning theory

According to the situated learning theory (Lave and Wenger, 1991) and the community of practice (CoP) (Wenger, 1999) deriving from the social theory of learning, learning is a “social participation”(Wenger, 1999, p. 4). Participation was “the social experience of living in the world in terms of membership in social communities and active involvement in social enterprises” (Wenger, 1999, p. 55). From a social-cultural perspective, English teachers construct their PIs by interpreting institutional expectations and interacting with significant others (Cheng and Starks, 2002) dynamically within certain institutions. The construction of PI is also the negotiation of meanings which occurs through a duality of participation and reification (Wenger, 1999). For participation, teachers actively participate in the practices of their working communities, thereby constructing PIs and acquiring knowledge of teaching (Clarke et al., 2014). For reification, teachers give external forms (such as teaching behaviors) to their internal teaching experiences (Wenger, 1999).

Three trajectories (peripheral, inbound, and outbound) (Wenger, 1999) of the participation in the CoP describe participants’ PI construction and reactions to PI tensions. Individuals following the peripheral trajectory stayed at the marginal position of the CoP and never engage in full participation. Individuals following the inbound trajectory positively engage in the community practices and anticipate a full membership. Members following the outbound trajectory leave the current community and move towards another outside group. This movement is activated by members’ development of new relationships, anticipation of future proposed roles, and viewing the world and themselves from new perspectives (Wenger, 1999).

### 3.3 Self-discrepancy theory

Drawing on the self-discrepancy theory (Higgins, 1987, 1989), there are three dimensions of teachers’ identification of self-concepts: ideal self (personal expectations), ought self (institutional standards), and actual self (actual performance) (Ferguson, 2011). They are also teachers’ beliefs or definitions of professional images in the past, at present, and in the future (Hamman et al., 2010).

In the construction of professional images, discrepancies between two self-concepts produce emotional issues or psychological discomfort (Higgins, 1987). The discrepancy between actual self and ideal self will result in dejection-related emotions (disappointment, dissatisfaction, shame) (Schlechter et al., 2022). The discrepancy between actual self and ought self will engender agitation-related emotions (self-contempt, fear, guilt) (Higgins, 1987). To overcome those negative emotions, teachers will enact agency to reconcile three selves iteratively through practice evaluation and combined personal and environmental factors (Ruan and Toom, 2022).

## 4 Methodology

This study is a narrative inquiry (Clandinin and Connelly, 2000) which applied in-depth interviews to collect data. Metaphorical biographies and thematic analysis were employed to analyze why novice English teachers experienced PI tensions, what are its manifestations, and how they navigated it.

### 4.1 Participants

The purposeful sampling strategy (Trotter, 2012) was employed to recruit three participants who fit the criteria: They were English teachers in the first stage (1–3 years) of their academic careers from three different private universities in China. Participants’ demographic information is presented in Table 1.

**Table 1:** Demographic information of participants.

Pseudonym	Gender	Teaching experience (year)	Location of workplace	Status
Ruby	Female	2	Middle China	Resigned and PhD candidate
Pam	Female	2	Southwestern China	Resigned and PhD candidate
Lucy	Female	2	Eastern China	On the job

### 4.2 Data collection

This study employed the in-depth interview and self-narration to collect data because teachers made sense of their PIs by narrating their experiences of PI tensions (Clandinin and Rosiek, 2007), especially the changes of emotions, attitudes, beliefs, behaviors, and motivations (Kılıç and Cinkara, 2020) in 2 years of teaching. The self-narrations were conducted 1 month after the in-depth interviews at the end of participants’ second year of teaching.

In in-depth interviews, participants answered several open-ended questions (see Table 2) in their teaching experiences based on a semi-structured interview outline (Kvale and Brinkmann, 2009) (see Table 3) covering effect factors of teachers’ identity construction (Zhang, 2016). Those factors were categories into five dimensions (cognitive, motivational, individual, institutional, social) belonging to two levels (personal, contextual).

**Table 2:** Open-ended questions (examples).

Periods	Questions	Effect factors of teachers’ identity construction (Zhang, 2016)
1st year of teaching	What is your understanding of being an EFL teacher? What kind of teacher did you consider yourself are in work? What difficulties were you confronted with? How was your relationship with students?	Belief Self-evaluation Difficulty and workload Student
2nd year of teaching	How did you conduct professional learning in teaching and academics? How did you improve your teaching ability? Do you think you have become the ideal teacher in your expectation? If not, what do you think the gap was? How did online teaching affect your professional identities?	Learning motivation Ability Expectation Educational reform

**Table 3:** Interview outline.

Effect factors of teachers’ identity construction (Zhang, 2016)		
Personal	Cognitive	Attitude; belief, knowledge; self-evaluation; expectation; ability
	Motivational	Learning motivation; career choice and plan
Contextual	Institutional	Working environment; value, culture, tenet, policy; support and job satisfaction; student; difficulty and workload; leader and colleague
	Social	Educational reform

In self-narrations, participants narrated their unforgettable critical incidents of PI tensions that greatly impacted their PIs in a narrative outline (see Table 4) designed according to three categories: images in crisis, experiences of crisis, and solutions to crisis (Sadovnikova et al., 2016). The length of each self-narration was between 1–1.5 h. Participants’ oral narrations were recorded and transcribed verbatims.

**Table 4:** Narrative outline.

Categories of professional identity crisis (Sadovnikova et al., 2016)		
Images in crisis	Experiences of crisis	Solutions to crisis
Professional image	Negative emotions	Actions to overcome PI tensions
Self-image	Changes of professional value, belief, and attitude	
	Self-reflection	

Teachers narrated their experiences of PI tensions in the form of ontological narratives (Somers and Gibson, 1994; Søreide, 2006) to make others understand their feelings of being novice EFL teachers at private universities, they also negotiated meanings and reconstructed PIs through narratives (Lieblich et al., 1998). Participants made sense of PIs by narrating their lived experiences of PI tensions through time and the author understood that process synchronously (Clandinin and Rosiek, 2007).

### 4.3 Data analysis

Data were analyzed in two approaches. The first approach is writing metaphorical biographies based on participants’ storied data. Biographies with metaphors are thick descriptions (Lichtman, 2012) presenting “detail, context, emotion, and the webs of social relationships” that connect participants with readers (Denzin, 1989, p. 83). Such a rhetorical reprocessing of participants’ accounts was a productive practice of writing as method of inquiry and analysis (Clandinin and Connelly, 2000). The creation of metaphors reflected a creative analytical process (CAP) (Richardson, 2000) combining traditional narration with literature tactics (Richardson, 2005). It aims at achieving an engaging presentation of findings and a resonating, thought-provoking effect of data analysis (Richardson and St. Pierre, 2005). Metaphors disclosed implications behind participants’ tension experiences (Lakoff and Johnson, 2003) and expanded the knowledge of novice teachers’ PI tensions from a social scientific perspective (Farquhar and Fitzsimons, 2013).

The process of creating those metaphors is recursive and reflective. Metaphors were revised and polished several times drawing on the heuristic standards (Sword, 2019). Each metaphor was chosen according to the participant’s traits. Thus, those metaphors foreground the uniqueness of participants and reflected their authentic feelings and thoughts. Based on different metaphors, the author tried to structure various modes of how novice EFL teachers reconstructed their PIs through navigating PI tensions and find similar patterns in their narratives as required in the narrative analysis (Polkinghorne, 1995). The features related to PI tensions were highlighted in participants’ work reality so that readers and the author were reminded that the emotions and meanings entailed in those metaphors were true (Lakoff and Johnson, 2003).

The author reflected on the subjectivity of her writing to ensure its academic value and effectiveness. Subjectivity was a critical representation of her thoughts as a researcher. While writing biographies, sensual and emotional data such as sympathy were generated (Richardson and St Pierre, 2005) and the author stepped deeper into participants’ inner worlds. The author felt responsible to speak up for those teachers in a marginalized group so that they would be better supported for future professional development. To guarantee the trustworthiness of biographies, the author revised and polished the initial drafts based on participants’ evaluations about whether her writing reflected their authentic feelings and thoughts.

Based on principles of creative analytical process (CAP) ethnography (Richardson, 2005), to ensure the consistency of data from the “writing process” to the “writing product” (p. 478), the author shared all recordings, transcripts, and biographic stories with participants for proofreading and member checking to ensure the authenticity of data analysis since it lies in “the confirmation by the participants of their reported stories of experience” (Webster and Mertova, 2007, p. 99). Participants’ further advice for improvement of the writing was also valued so that the creative writing would truthfully reflect participants’ genuine thoughts. After all interviews, if there was any ambiguity during data analysis, the author maintained audit trails and added questions to acquire more information from participants.

The second approach of data analysis was deductive thematic analysis (Braun and Clarke, 2006) applied to explicate meanings behind teachers’ narrations of their PI development. The analysis begins with a theoretical framework consisting of three theories (social identity theory, situated learning theory, self-discrepancy theory) and existing literature (influencing factors of teachers’ PI construction) (Mohamed, 2020). The coding and data analysis is guided by a pre-established hypothesis that the PI tension of novice EFL teachers is caused by discrepancies among three self-concepts and appears as a conflict among three selves. Participants’ narratives were read thoroughly, compared iteratively, and classified into codes, initial and potential themes according to the analytical process of reflexive thematic analysis (Braun and Clarke, 2022). Data was analyzed and codes were extracted based on three topics: causes, manifestations, and solutions to PI tensions according to the research questions. Table 5 present the correspondence between three theories and three topics:

**Table 5:** The correspondence between theories and topics.

Theoretical framework	Three topics in data analysis		
	Causes of PI tensions	Manifestations of PI tensions	Solutions to PI tensions
Social identity theory	Social comparison	Social categorization Social identification	Social change; social mobility
Situated learning theory	Peripherality		Inbound, peripheral, and outbound trajectories; situated learning
Self-discrepancy theory	Discrepancies among three self-concepts	Conflict among three selves	Reconcile three selves

The thematic analysis followed the three-dimensional narrative space framework (Clandinin and Connelly, 2000; Clandinin, 2013) to “position inquiries within” (Clandinin and Connelly, 2000, p. 120) participants’ lived stories and think narratively. Clandinin and Connelly (2000) cited Deweyan’s ontology of experience including two criteria (interaction and continuity) and the concept of situation (Dewey, 1938) to interpret narrative inquiry as the theoretical foundation of their notions. Dewey’s theory also served as an important theoretical foundation for data analysis including three features: relational, temporal, and continuous, which were manifested within the narrative form (Dewey, 1938).

The coding process was firstly completed by the author and then checked by a third party (her PhD supervisors) in a process of data interpretation and analysis to assure readers of trustworthiness. Moreover, since interviews and narratives were recorded and transcribed in Chinese, the original data needed to be translated from Chinese to English for analysis. To ensure the accuracy of the translated versions, the author asked a translator as an external auditor to proofread the translated English versions.

The triangulation strategy (Denzin, 2007; Neale, 2019) was used to ensure the trustworthiness of this study. Methods of data collection (interviews and self-narrations) and sources of data are multiple to guarantee the credibility (the truth of data) and seek corroboration and integrity of findings (Greene et al., 1989). Two approaches of data analysis (metaphorical biography and thematic analysis) were also conducted to analyze findings in an elaborate and deep way, thus gain a comprehensive understanding of teachers’ PI tensions (Patton, 1999).

## 5 Findings

Metaphorical biographies and thematic analysis were intertwined in data analysis. Meanings within codes and themes were likened to concrete objects in biographies in the analytical framework consisting of three topics (manifestations, causes, and solutions to PI tensions). Those two approaches were conducted simultaneously. Codes and themes were extracted from participants’ narrations, meanwhile they were presented and analyzed in three metaphorical biographies with illustrations about participants’ tension experience.

### 5.1 Walking through the tunnel—biography of Ruby

**Figure 1:**
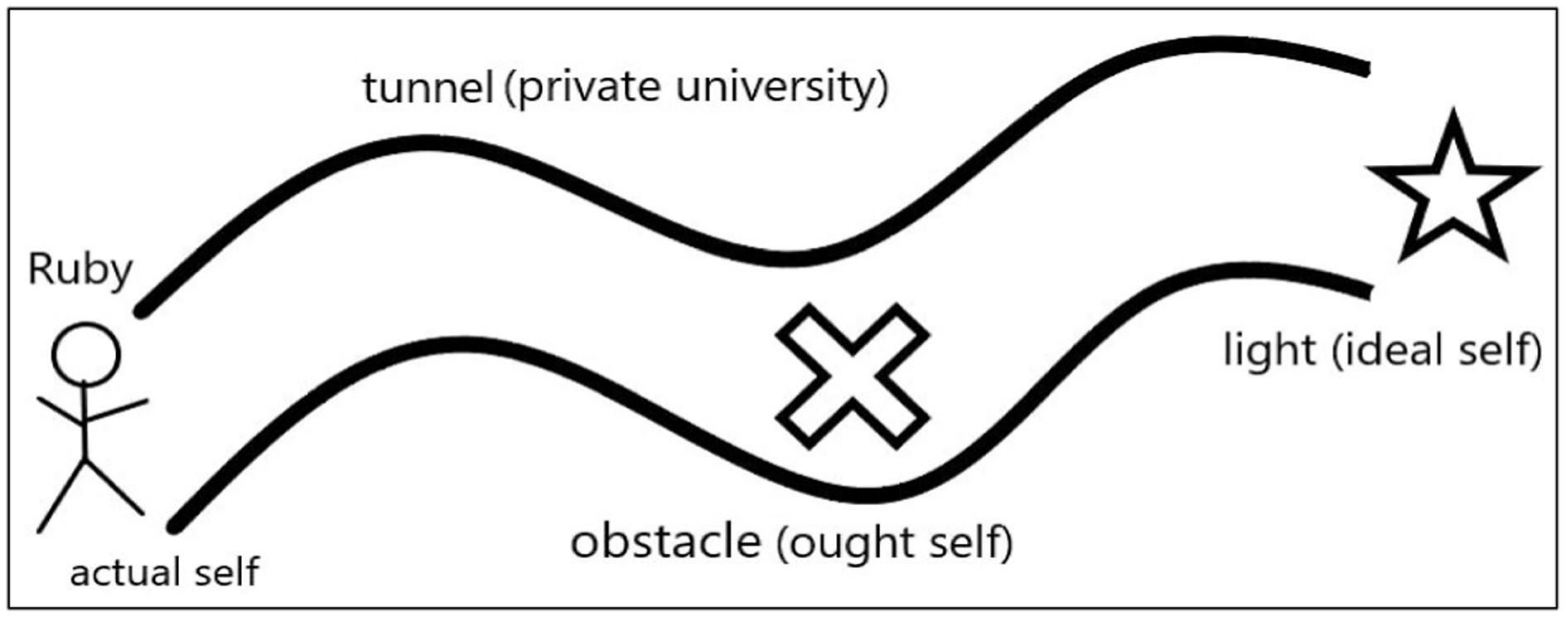
Biography of Ruby.

Ruby’s experience of PI tensions was marked by a reconciliation of three self-concepts, as shown in Figure 1. Her private university was likened to a tunnel. Her ought self was like an obstacle inside the tunnel. Her ideal self was like a light directing her way out of the tunnel.

Ruby was diligent and dedicated. She had a great passion for learning and teaching English. In the first year of work, Ruby’s PI tension was manifested in a gap between her actual performance and the standards of the ought self. She started teaching with a preconceived belief that a teacher’s elaborate lecturing was essential for students’ learning. So, she spent most time in class imparting knowledge to students. Her class was dull without many interactions with students and the teaching outcomes were inefficient. Ruby received negative evaluations from students. Then, she experienced a low level of teaching efficacy. To improve performance, in the second year, Ruby adjusted her teaching style and added English videos and broadcasts to her teaching content. In class, she encouraged students to participate in class activities in a lively tone. Later, her students were more focused in class and gave her positive evaluations. Ruby acquired a sense of achievement and an increased teaching efficacy. Thus, she met the standard of the ought self: a qualified English teacher.

However, Ruby was not satisfied with only achieving her ought self, she expected to realize her ideal self-image: a teacher skillful at both teaching and academic research. Novice teachers were managed strictly but not offered enough academic supports at her university. Ruby believed that she could never achieve her academic ambition if she just fulfilled the duties prescribed in the ought self. So, her ought self-concept became an obstacle of her PI construction.

The manifestations of Ruby’s PI tension were diminished autonomy and work commitment, and a feeling of job insecurity. Ruby followed a peripheral trajectory and did not complete the full participation at her university (Lave and Wenger, 1991). She adopted a social mobility mindset and was ready to leave at a proper time (Wenger, 1999). In the third year, Ruby navigated the PI tension by resigning from her university to pursue a PhD for a higher social status (Tajfel and Turner, 1986). She walked out of the tunnel directed by the aspiration of realising the ideal self.

### 5.2 Swimming against the stream—biography of Pam

**Figure 2:**
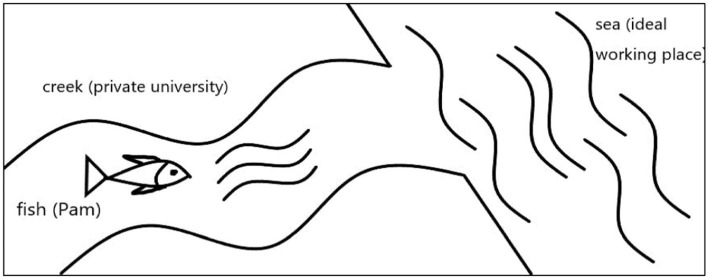
Biography of Pam.

Pam’s experience of PI tension was about a struggling adaption to an unfavourable working context, as shown in Figure 2. Her private university was likened to a creek where she swam like a fish against the stream, just like she resolved difficulties in teaching. Pam could not realize her career goal at the creek, so she swam towards the sea for an ideal working place.

Pam was sedate and meticulous. She was longing to become a teacher. However, after employment, her confidence and authority were diminished due to setbacks in teaching, that led to her PI tension. In the first semester, Pam implemented a strict teaching method in class, but some students did not cooperate with her. Pam endeavored to teach *College English* well. But students lacked the learning motivation, and the course was undervalued by the university. Pam also felt inadequate in writing syllabuses for academic courses because of the difficult teaching contents. Students were not interested in them, and the teaching effects were unsatisfying.

Pam’s manifestations of PI tension were self-doubt and frustration. She doubted her competence because she received negative evaluations from students. To overcome tensions, Pam learned teaching skills from her colleagues. In the second and third semesters, Pam adjusted her teaching method and content based on students’ needs, language proficiency, and learning habits. She designed games in class to arouse students’ interest of learning and asked students to finish most homework in class. Afterward, students’ learning effect was improved. Pam acquired respect and gratitude from students and regained confidence.

However, she did not engage in full participation at her department (Wenger, 1999) because she clashed with her leaders over the working style. Pam believed that her actual self was far away from her ideal self because she could not fulfill the ambitions of wining prize in teaching and publishing academic papers due to limited institutional supports. It’s like the converse stream impeded the fish’s advancement. Therefore, Pam resigned and studied abroad for a PhD. She adopted the social mobility mindset to search for a new working context (Tajfel and Turner, 1986).

### 5.3 The sun in winter—biography of Lucy

**Figure 3:**
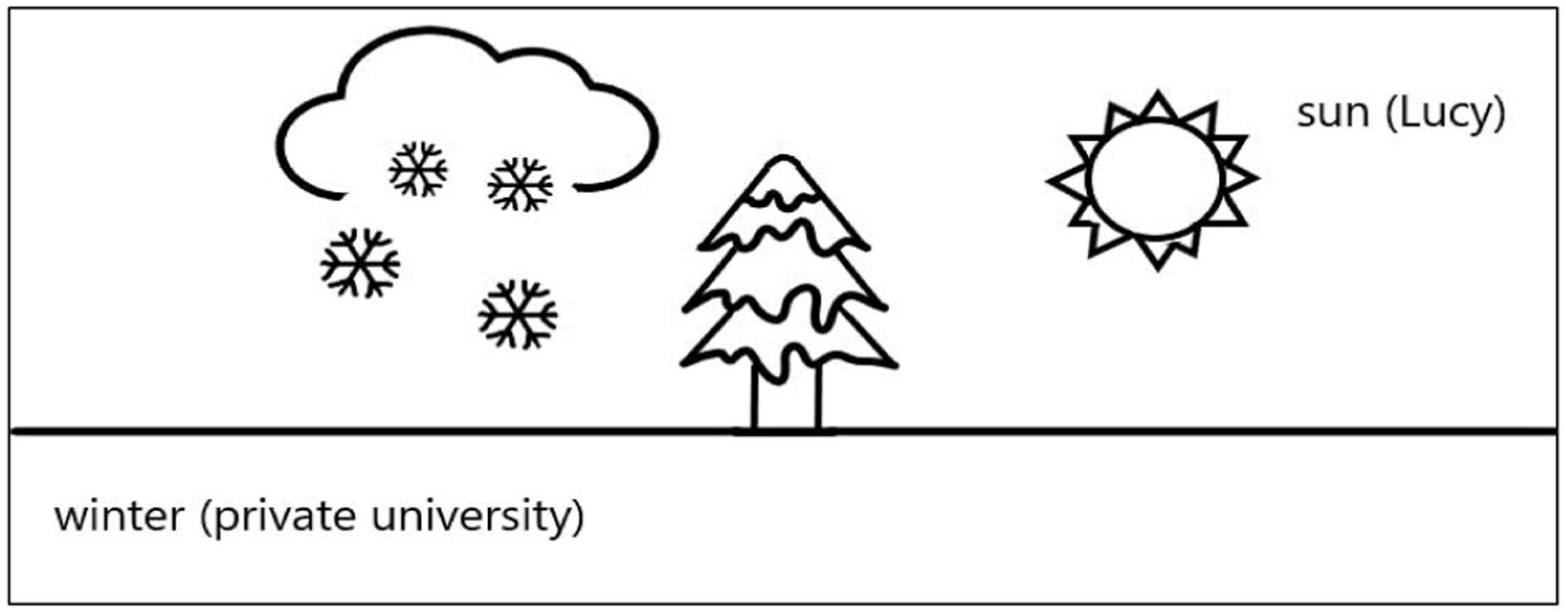
Biography of Lucy.

Lucy’s experience of PI tension was about the exertion of self-agency to contribute to teaching, as shown in Figure 3. Lucy was like the sun bringing warmth to her private university, although it was a harsh, less supportive working context like the winter.

Lucy was an ambitious and versatile teacher. Her passion for teaching English was likened to the energy of the sun. The trigger of Lucy’s PI tension was the heavy teaching and administrative workload. Lucy was assigned to many courses in one semester including a difficult course about writing trade documents in English. She was unsatisfied with her teaching performance because of limited time for preparation. She also lacked time and supports for doing academic research. Lucy expected to become a teacher excellent in both teaching and research. The unfulfilled researcher role caused her self-doubt.

The manifestation of Lucy’s PI tension was the lack of self-worth because she did not achieve the ideal self. Lucy applied her teaching value and philosophy to solve her PI tension (Nickel and Crosby, 2022). She tried to realize her ideal self by increasing the work commitment. She cared much about the practicability of knowledge and students’ future employment. Lucy strove to cultivate competitive, versatile talents who acquired both language skills and knowledge in an industry. She initiated an online teaching project about applying computer-aided translation to the trading industry. Her action improved the teaching quality at her university and increased her confidence and self-worth.

The winter needed the light and heat from the sun. Lucy’s working context was promising to be improved thanks to young, dedicated teachers like her. The retention of teachers would be ensured if the context was improved.

### 5.4 Thematic analysis

Besides the metaphorical descriptions of novice teachers’ PI tension experiences, for a deep and focused interpretation of their tensions within the theoretical framework, meanings in their narratives were distilled into codes and themes in three topics: causes, manifestations, and solutions to the PI tension. Table 6 presents these codes and themes:

**Table 6:** Themes and codes in three topics of PI tension.

Topics	Themes	Codes
Causes	Role conflict	Conflicting multiple roles; lacking time for research; teaching workload; transition from a student to a teacher
	Setbacks in teaching	Uncooperative students; unsatisfying teaching outcomes; negative evaluations from students
	Professional incompetence	Knowledge obsolescence; lacking autonomy; lacking effective teaching skills
	Development limitation	Lacking institutional support; unfavourable working context; unattainable academic ambitions; pressure from social evaluation
Manifestations	Impaired professional identification	Lacking sense of achievement; frustrated self-esteem; lacking self-worth; low teaching efficacy; lacking authority; negative emotions
	Unfulfilled organizational identification	Lacking sense of belonging; low organizational commitment; disapproval of GI
Solutions	Reconciling three selves	Situated learning; adjusting teaching methods, acquiring knowledge; attending competitions; pursuing a PhD; changing the working context
	Institutional supports	Efficient training; collaborative cop; academic supports; improving salary, welfare, social status and reputation

There are four themes about causes of PI tension: *role conflict*; *setbacks in teaching*; *professional incompetence*; *development limitation*. First, participants mentioned that three roles (academic, teacher, administrator) were always in conflict (Beijaard et al., 2004) because they failed to balance tasks inherent in those roles. Lucy said:

I was assigned loads of different administrative tasks besides teaching several courses…I felt tired switching between multiple roles. (L-I1).

Three roles were ranked differently by participants in the salience hierarchy (Brenner et al., 2014). The academic role was most salient, followed by the teacher role and the administrator role. Participants considered research as an inherent demand in their PIs and making academic achievements as an important approach to realize the ideal self. However, they were exhausted with administrative tasks and teaching but had little time conducting research. The fulfillment of two less salient roles impeded the fulfillment of the most salient role, which impaired their self-worth.

The second theme is *setbacks in teaching*. Students influenced novice teachers’ PI construction greatly. Participants tried to avoid negative evaluations from students because they had “survival concerns” (Fuller and Bown, 1975) in the early-career period. They felt hard to adjust teaching methods aligning to students’ competence and meanwhile ensure positive evaluations. Sometimes their students were uncooperative in class if they could not understand the teaching contents or disliked teachers’ methods and styles. Then, the teaching outcomes would be ineffective, and students might give teachers negative evaluations. Lucy said:

I felt anxious about my choice of teaching methods. Should I let students study easily, so that I could have positive evaluations, or should I have strict requirements on them so that they could really learn knowledge? (L-I2).

Negative evaluations affected many aspects of their PI, thus caused the PI tension (Hutchins and Rainbolt, 2017). In Ruby’s first year of teaching, her self-efficacy was deteriorated and she felt a deprival of autonomy and authority because of unsatisfying teaching outcomes and negative evaluations from students. Pam’s confidence and authority were undermined due to the non-cooperation of students in a class test. Afterward, she worried about her relationship with students and doubted her competence. She said:

I became more cautious and assigned less homework to students than before…I also adjusted some of my teaching contents…I had self-doubt if I was fit to be a teacher. My teaching and communication abilities were not as good as I thought. (P-I1).

The third theme *professional incompetence* reflects that participants perceived that their knowledge were outdated and teaching skills were insufficient. They felt much pressured due to the gap between their competence and students’ high requirements for them. Lucy taught business English, but sometimes she could not explain to students about the latest English expressions in the international trade industry. She also worried about teaching an unfamiliar course.

Participants’ lack of pedagogical knowledge and unfamiliarity of students’ learning habits led to unsatisfying teaching results. They underwent a reality shock due to the gap between the presumption of what to teach and students’ actual needs (McIntush and Garza, 2023). In Pam’s class, students were not interest in her explanation of abstract terms. She said:

I doubted my professionalism when I wrote the syllabus and taught the course *Thesis Writing*…I thought maybe my knowledge was not enough, and I became a little confused and anxious. (P-I2).

The fourth theme *development limitation* denotes that the professional development of participants was restricted in unsupported working contexts where they could not attain their academic ambitions. This theme echoed with the previous finding that private universities in China have not provided ideal academic environments for young teachers (Wang et al., 2020). Ruby disliked her working atmosphere where the faculty did not form a collaborative team. That impeded her establishment of the group membership. Pam’s department did not offer her many opportunities to hone the research skills, so her confidence flagged, and she felt less committed to work. Lucy was assigned to teach courses she was not good at, that made her unable to combine teaching with research to achieve her ideal state.

Participants worried about their career development at private universities because of social negative evaluations. The public regarded teachers at private universities as not qualified enough with limited teaching experience (Lei, 2012). They were lower paid and subordinate in comparison to their public university counterparts (Zhou et al., 2018). Ruby sensed a job precarity after witnessing a high turnover rate of faculty at her department. That became the fundamental reason of her PI tension. She said:

I have been thinking about resignation…At a private university, I always have the feelings of crisis, uncertainty, and insecurity. (R-I1).

The manifestations of PI tension among participants were distilled into two themes: *impaired professional identification* and *unfulfilled organizational identification*. The first theme *impaired professional identification* implied that many aspects of teachers’ PI were impaired. Ruby said that her interactions with students did not reach her expectation, which decreased her sense of achievement and commitment to teaching. This finding proved that teachers intensified PIs by their commitment to contacting and working with students (van Lankveld et al., 2017). Poor teaching effects resulted in participants’ frustration and anxiety. Pam felt hard managing the class, which frustrated her self-esteem. Lucy perceived a low teaching efficacy because she had to offer several different lessons to students in a semester and she was unfamiliar with the teaching contents.

The second theme *unfulfilled organizational identification* indicated that participants lacked the job satisfaction and work commitment in dissatisfactory working environments. Ruby’s sense of belonging diminished because the administration of her university was problematic and the faculty was not cohesive. Pam worked devotedly but did not receive corresponding approval from the leadership. She felt like a marginal person excluded from the teaching and research teams and had no sense of membership. That resulted in negative emotions affecting her performance in daily work. She said:

My university leaders did not care about the department of applied language where I worked…The department and I were unvalued. (P-I1).

There are two themes about solutions to PI tension: *reconciling three selves* and *institutional supports*.

First, participants navigated the PI tension by reconciling three self-concepts. The discrepancy between the actual self and the ought self appeared when participants’ teaching performances did not reach institutional requirements. They reconciled those two selves by situated learning from model teachers, adjusting teaching methods, and acquiring interdisciplinary knowledge. Participants demonstrated the reflective and agentive thinking (Priestley et al., 2015) as they constantly evaluated and improved their teaching practices built upon the knowledge learned from model teachers. When participants performed their prescriptive teacher roles well, they achieved self-verification and increased their self-esteem and self-efficacy (Burke and Stets, 2009). Pam altered her prior teaching methods after she observed the lessons of a leader who designed effective activities to attract students’ attention. Ruby reflected on her performance then adjusted her teaching beliefs. Afterward, she regained confidence and the sense of authority. She said:

What makes a teacher popular are interactive and flexible teaching methods…The teaching content should not be scripted. (R-I1).

Lucy upgraded her teaching ability by acquiring interdisciplinary knowledge in international trade from one student. That strengthened her relationship with students and her teaching efficacy.

After participants achieved the ought selves, if they believed that their prescriptive images were far from their expected professional images, the discrepancy between the ought self and the ideal self arose. Participants would either adopt the social change mindset to upgrade the in-group status (Abrams and Hogg, 1990) like Lucy or adopt the social mobility mindset to leave their ill-fitted working contexts and pursue academic achievements like Ruby and Pam. Lucy delved into approaches to improve the education quality of English major and persisted in self-learning. Her actions promoted the welfare of her professional group (Ndoja and Malekar, 2020). Ruby and Pam perceived that their academic ambitions could not be realized at private universities, so they resigned and pursued a PhD to be able to work at a public university after graduation.

Second, participants expected institutional supports to navigate PI tension. Lucy expected sufficient guidance of publishing papers to upgrade her academic qualification. Rudy longed for a cohesive community where she could acquire a collective sense of honor. Pam wished for increased salary and welfare to ensure her work commitment. She said:

What drives me to work is the desire to make achievements in teaching or research. I expect targeted academic lectures, project funds, and pre-employment trainings. (P-I2).

A supportive professional group would ensure participants competitive social status and strengthen their sense of belonging (Tomo, 2019). Enhancing the prestige of the private university would promote participants’ organizational identification (Liu et al., 2014).

## 6 Discussion

Based on a sociocultural theoretical framework, participants’ PI tension were caused by the conflict between the ETI and the PUTI. To navigate tensions, participants adopted two approaches: assimilation and dissimilation. A reciprocal relationship between teachers and private universities is suggested to support participants’ professional development.

### 6.1 The conflict between the ETI and the PUTI

In the social identity theory, participants’ PIs were also social identities or group identities (GIs) because they belonged to two social groups: English teachers and private university teachers. Social identities stand for essential components of teachers’ self-definitions that existed within organizations or situations (Fisher, 1986). They were situation-related and formed in interactions with students and colleagues based on “values, beliefs, norms, and demands inherent in the identities” (Ashforth and Mael, 1989, p. 29). The conflict between two GIs was presented by discrepancies among three self-concepts.

Participants’ construction of GIs was accompanied by ambivalence and predilection. They held ambivalent attitudes towards two GIs because they were endowed with opposite social evaluations between groups (Tajfel and Turner, 2004): the social evaluation of the private university teacher group was negative and the social evaluation of the English teacher group was positive.

First, for the PUTI, participants disapproved of this group’s worth and held negative attitudes towards this in-group membership due to a negative social comparison with the out-group (public university teacher) membership (Islam, 2014). Consequently, participants’ self-esteem was frustrated (Ashforth and Mael, 1989) and they could not fully internalize the values and standards of the private university communities (Gökçe, 2020). Second, participants had a predilection for the ETI. They regarded the ETI as a more valued identity than the PUTI based on their salience hierarchy of multiple identities (Brenner et al., 2014). So, they always defined themselves as English teachers and activated this identity regardless of the working contexts (Stets and Burke, 2000).

Participants encountered the PI tension because the development of their ETI were limited at private universities. The market-oriented, interdisciplinary trends of ELT in China have set higher requirements for novice English teachers (Murray et al., 2023; Xu and Knijnik, 2021). Participants were burdened with tasks of delivering high-quality English courses to cultivate interdisciplinary talents (Dai and Lin, 2021). However, they perceived that both English teachers and the English discipline were in unvalued statuses at private universities. Participants felt less competitive than teachers at other departments, and were afraid of being substituted, especially during and after the COVID-19 pandemic when online English learning materials were abundant and easily accessible (Shadiev and Yang, 2020; Zhang and Perez-Paredes, 2021). The ETI was predominant by which participants were motivated to work and acquired a sense of achievement (Stets and Burke, 2000). When the development of this identity was impeded by the PUTI, participants’ motivation and work commitment would decrease.

### 6.2 Two approaches of navigating the PI tensions

**Figure 4:**
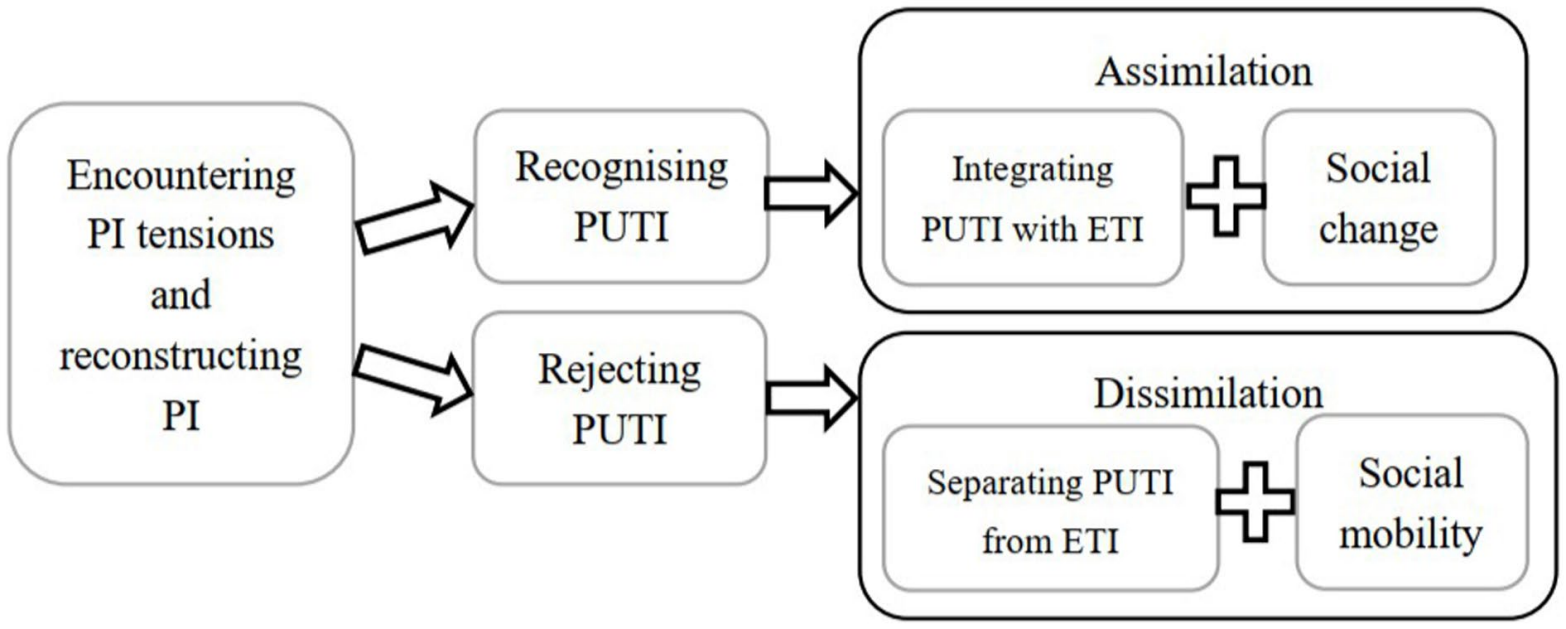
Two approaches of navigating the PI tensions.

Participants adopted two approaches to navigate the PI tensions: assimilation and dissimilation, as shown in Figure 4.

After participants encountered the PI tensions, they reconstructed their PIs through reconciling three selves and solving the conflict between two GIs. In this process, they formed two approaches to navigate tensions based on two opposite attitudes towards the PUTI: if participants recognized their PUTIs, they would choose the assimilation approach; if participants rejected their PUTIs, they would choose the dissimilation approach. The aim of both approaches was sustaining and improving their self-esteem to pursue a positive self-concept (Tajfel and Turner, 2004).

Lucy adopted the assimilation approach. She integrated the PUTI and the ETI. Lucy followed the inbound trajectory in which she moved from the peripherality to the center position in interactions with the contexts (Liu and Xu, 2013). Lucy gained the in-group membership by adapting to the working environment and increasing the work commitment. When Lucy perceived her in-group membership inferior to the out-group membership, she adopted the social change mindset (Tajfel and Turner, 1986). Lucy upgraded her in-group status by improving the education quality at her private university.

Ruby and Pam adopted the dissimilation approach. They separated the PUTI from the ETI. They followed the peripheral trajectory in which they stayed at the peripheral status and failed to fit in the center communities. They could not reconcile their teaching beliefs with institutional values, so they did not complete the social identification at their private universities, which resulted in their lack of sense of belonging (Lee and Robbins, 1998). They felt frustrated and undervalued because their academic development was restricted. Later, they followed the outbound trajectory and resigned from private universities to pursue the PhD degree. They adopted the social mobility mindset (Tajfel and Turner, 1986) in which they planned to move to public universities with a higher social status compared to the private university.

### 6.3 Achieving a reciprocal teacher-university relationship

Findings implied that a reciprocal and symbiotic relationship between the private university and teachers would facilitate the development of both parties. Such a relationship will address teachers’job insecurity and improve their mental health and well-being (Van der Vyver et al., 2020).

Nationally, policy makers are suggested to offer a well-established identity policy to accustom teachers to their new work order and reconstruct proper identities to participate and be involved in updated contexts (Bolívar et al., 2014). They are also supposed to provide more research funding for private universities, enabling novice teachers to fulfill their academic aspirations there and thus meet the standards of their ideal selves. In this way, they can have a stronger sense of identity with their status as private university teachers, remain loyal to their posts, and contribute to the further teaching and academic development of private universities. On the university side, if private universities want to retain novice teachers, they could offer teachers more academic, emotional, and economic support because the organizational support is a facilitator of teachers’ academic development (Falola et al., 2020).

First, the premise of participants’ work engagement with passion was sufficient support in their principal tasks—teaching and academic research. They expected an efficient teacher management system and an incentive mechanism by which they could be encouraged to spend more time on teaching and research, which facilitated their academic development (Yao, 2022). Participants needed the guidance from experienced, model teachers to adapt to the working context and relied on cooperation with colleagues to solve problems in teaching and managing students. They also anticipated customized assistance based on their research interest. When they performed the teacher and academic roles well, they would realize their self-worth.

Second, emotional support is necessary. An encouraging working atmosphere could help teachers overcome negative emotions triggered by the PI tension. A supportive, collaborative professional community is a crucial source of emotional support for teachers to recognize their GIs and acquire a sense of achievement, it also facilitates their cognitive learning of knowledge and skills in academic research (Yuan et al., 2022). Participants expected respect from institutions because it would motivate their job engagement. The stronger teachers were motivated to teach, the more satisfied teachers felt with their jobs, the more dedicated to the work they would be (Nayir, 2012).

Participants also expected more agency and autonomy in deciding teaching methods and the evaluation criteria to overcome tensions in PI construction (Adada, 2023), so that they could form a positive attitude towards their jobs and enhance the group membership. From an ecological viewpoint, teachers’ agency results from a constantly unique interplay between individuals’ potential and the social and material contexts in which they act (Farmasari, 2021). If the contexts are supportive and beneficial, novice English teachers’ agency and potentials will be facilitated. They may achieve“continuing development” which offers “valuable opportunities for reflections, spontaneity, and innovations” (Yuan et al., 2022, p. 12) in teaching.

Third, participants anticipated more economic support. Private universities are suggested to raise teachers’ salaries and upgrade their welfare and benefits since the increase of teachers’ earnings will improve their social status (Hanushek et al., 2004).

The other side of the reciprocal relationship between teachers and private universities is that teachers can contribute to the development of universities. Participants are suggested to conduct organizational citizenship behaviors (Eres, 2010), such as contributing to the education quality and management of private universities and making academic achievements. These behaviors will improve the professional qualification of the teacher groups, which will further upgrade the reputation and status of private universities. Then, teachers’ fame and social status would be improved. They would approve of their social groups and have a strong sense of belonging.

## 7 Conclusion

This study explored the causes, manifestations, and solutions to the PI tension of three novice English teachers at China’s private universities. Data analysis contributed new insights to the social identity theory that the development of one GI (ETI) can be impeded by that of another GI (PUTI). Whether teachers would strengthen the sense of belonging depended on whether they approved of their GIs or not. This study also contributed to the knowledge of the situated learning theory. Besides the inbound trajectory, teachers’ participation in the CoP of private universities was marked the peripheral and outbound trajectories in which they anticipated an out-group membership to overcome the PI tension and consolidate their PIs. In terms of the self-discrepancy theory, the causes of participants’ PI tension were a disunity of two GIs and the discrepancies between three self-concepts.

Metaphors explored positive connotations of teacher–context interactions and implied a symbiotic and reciprocal teacher-university relationship. Novice English teachers depended on the private university to hone teaching skills and learn to become qualified teachers. They bore the responsibility to make contributions. Private universities relied on novice teachers to improve the education quality. They could offer teachers supportive, promising working contexts to help them survive the first 3 years and realize their ideal images.

This study has some limitations. First, it focused on a particular context and a specific group of teachers. Therefore, findings cannot be replicated and generalized for every teacher in all types of education institutions. The second limitation is that the data are participants’ self-narrations. More field notes of teachers’ lecturing, class management, and their interactions with significant others could be collected to acquire a comprehensive understanding of their PI construction in interactions with contexts (Kaya and Dikilitaş, 2019) and an extensive interpretation of their PI tensions. Teaching records and plans could be added as documentary evidences to corroborate participants’ oral accounts. In the future, more comparative studies could be conducted among teachers at different career stages, from different disciplines, at different education institutions. Future research could cover areas like teachers’ PI tensions in the digital education era and the influence of artificial intelligence on teacher PI.

## Data Availability

The original contributions presented in the study are included in the article/supplementary material, further inquiries can be directed to the corresponding author.
