# Correction: Leave or stay? A narrative inquiry of tensions in novice English teachers' professional identity construction at China's private universities

**DOI:** 10.3389/fpsyg.2025.1751061

**Published:** 2025-12-17

**Authors:** Xiangchen Zhang

**Affiliations:** 1 School of Humanities and International Studies, Zhejiang University of Water Resources and Electric Power, Hangzhou, China; 2 The School of Curriculum and Pedagogy, The Faculty of Arts and Education, The University of Auckland, Auckland, New Zealand

**Keywords:** narrative inquiry, novice English teacher, professional identity construction, tensions, private university

The reference for “Kaye, D. (2023). The effect of self-discrepancies on emotions and life satisfaction. Biomed. Sci. Clin. Res. 2, 94–99” was erroneously included in the article. This reference has been removed from the article, and its in-text citation has now been removed from Section 3 “**Theoretical framework**,” Subsection 3.3 “*Self-discrepancy theory*,” Paragraph 2.

Schlechter et al. (2022) was not cited in the article. The citation has now been inserted in Section 3 “**Theoretical framework**,” Subsection 3.3 “*Self-discrepancy theory*,” Paragraph 2 and should read:

“In the construction of professional images, discrepancies between two self-concepts produce emotional issues or psychological discomfort (Higgins, 1987). The discrepancy between actual self and ideal self will result in dejection-related emotions (disappointment, dissatisfaction, shame) (Schlechter et al., 2022).”

The reference for Schlechter et al. (2022) has been added to the references list.

The original version of this article has been updated.
